# Cytokines Induce Faster Membrane Diffusion of MHC Class I and the Ly49A Receptor in a Subpopulation of Natural Killer Cells

**DOI:** 10.3389/fimmu.2016.00016

**Published:** 2016-02-04

**Authors:** Sunitha Bagawath-Singh, Elina Staaf, Arie Jan Stoppelenburg, Thiemo Spielmann, Taku Kambayashi, Jerker Widengren, Sofia Johansson

**Affiliations:** ^1^Department of Microbiology, Tumor and Cell Biology, Karolinska Institutet, Stockholm, Sweden; ^2^Experimental Biomolecular Physics, Department of Applied Physics, Royal Institute of Technology, Stockholm, Sweden; ^3^Department of Pathology and Laboratory Medicine, Division of Rheumatology, Perelman School of Medicine, University of Pennsylvania, Philadelphia, PA, USA; ^4^Department of Medicine, Perelman School of Medicine, University of Pennsylvania, Philadelphia, PA, USA

**Keywords:** major histocompatibility complex, natural killer cells, molecular dynamics, cytokine, fluorescence correlation spectroscopy, plasma membrane, Ly49 receptors, molecular diffusion

## Abstract

Cytokines have the potential to drastically augment immune cell activity. Apart from altering the expression of a multitude of proteins, cytokines also affect immune cell dynamics. However, how cytokines affect the molecular dynamics within the cell membrane of immune cells has not been addressed previously. Molecular movement is a vital component of all biological processes, and the rate of motion is, thus, an inherent determining factor for the pace of such processes. Natural killer (NK) cells are cytotoxic lymphocytes, which belong to the innate immune system. By fluorescence correlation spectroscopy, we investigated the influence of cytokine stimulation on the membrane density and molecular dynamics of the inhibitory receptor Ly49A and its ligand, the major histocompatibility complex class I allele H-2D^d^, in freshly isolated murine NK cells. H-2D^d^ was densely expressed and diffused slowly in resting NK cells. Ly49A was expressed at a lower density and diffused faster. The diffusion rate in resting cells was not altered by disrupting the actin cytoskeleton. A short-term stimulation with interleukin-2 or interferon-α + β did not change the surface density of moving H-2D^d^ or Ly49A, despite a slight upregulation at the cellular level of H-2D^d^ by interferon-α + β, and of Ly49A by IL-2. However, the molecular diffusion rates of both H-2D^d^ and Ly49A increased significantly. A multivariate analysis revealed that the increased diffusion was especially marked in a subpopulation of NK cells, where the diffusion rate was increased around fourfold compared to resting NK cells. After IL-2 stimulation, this subpopulation of NK cells also displayed lower density of Ly49A and higher brightness per entity, indicating that Ly49A may homo-cluster to a larger extent in these cells. A faster diffusion of inhibitory receptors could enable a faster accumulation of these molecules at the immune synapse with a target cell, eventually leading to a more efficient NK cell response. It has previously been assumed that cytokines regulate immune cells primarily via alterations of protein expression levels or posttranslational modifications. These findings suggest that cytokines may also modulate immune cell efficiency by increasing the molecular dynamics early on in the response.

## Introduction

Natural killer cells play an essential role in innate immunity and protect the host against viral infection and tumors (1). Murine NK cells express a family of inhibitory receptors called Ly49 receptors, which have major histocompatibility complex (MHC) class I molecules as ligands. MHC class I is expressed by virtually all healthy cells, but can be downmodulated upon infection or in tumorigenesis. In the absence of sufficient cognate MHC class I expression, NK cells proceed to kill the target cells due to lack of signaling through their inhibitory receptors, a process called missing-self recognition (2).

Cytokines are important modulators of NK cell activity. Whereas IL-15 is important for NK cell survival, maturation, and priming, interleukin-2 (IL-2) and type I interferons are prototypical cytokines of NK cell activation (3, 4). IL-2 is currently used for clinical NK cell activation in cancer treatment (5). The increased cytotoxicity *in vitro* and *in vivo* induced by cytokines is dependent on the upregulation of numerous proteins, including several adhesion molecules, as well as effector molecules (4). Just a brief stimulation with IL-2 augments adhesion and cytotoxicity, primarily against missing-self targets (6). IL-2 also augments the NK cell dynamics at a cellular level. After several days in IL-2 culture, NK cells display a more migratory phenotype and a more dynamic migratory pattern (7). However, IL-2 stimulation may not affect all NK cells equally, since a minority of IL-2 stimulated NK cells were observed to perform the majority of kills (8). Type I interferons, such as interferon alpha and beta (IFN-α + β), are also strong inducers of NK cell cytotoxicity, primarily during viral infections (9, 10). Type I interferons, in addition, strongly upregulate MHC class I on many cell types, including lymphocytes (11, 12).

When IL-2 binds to its receptor, an association with the cytoskeleton is induced, and the diffusion rate of the receptor complex is slowed down (13). However, although much is known about the cellular dynamics in response to cytokines, very little is known about how cytokines affect molecular dynamics beyond its own receptor. This is despite the vital role of lateral diffusion of molecules within membranes for all diffusion-limited bimolecular interactions. Examples of such reactions are ample, and also involve reactions crucial for immune cell regulation and activation. For instance, lateral diffusion of receptors is responsible for the formation of micro-clusters and the subsequent immune synapse in T cells (14). The diffusion rate of ligands impacts the degree of T cell activation (15), and the activation of CD4 T cells is regulated by the diffusion rate of lck between the CD3 and CD28 receptors (16).

Apart from interacting with its ligands in *trans*, on target cells, Ly49 receptors also interact with their MHC class I ligands in *cis*, in the membrane of the NK cell itself. Such *cis* interactions prohibit Ly49 from interacting with MHC class I in *trans* (17). Thus, the total number of receptors that are “free” and, therefore, available to interact with MHC class I in *trans* is decreased by *cis* interactions. Since Ly49 receptors bound in *cis* do not signal negatively, the sequestration of receptors in *cis* limits the total inhibitory input that the NK cell can receive, consequently lowering the threshold for NK cell activation. *Cis* interactions are also suggested to be important for NK cell education, the process where NK cells are enabled to react on the lack of expression of self-specific MHC class I on target cells (18). The surface expression of MHC class I can affect the proportion of Ly49A that is bound in *cis*. In a transfected cell line, cells with a high surface expression of MHC class I displayed high proportions of Ly49A bound in *cis*, and vice versa (19). This is typical of a diffusion-limited bimolecular interaction in steady state. The molecular density within the cell membrane, as well as the diffusion rates of these proteins, are thus important factors in determining the rate of receptor–ligand interactions between Ly49A and H-2D^d^ in *cis*. This could ultimately be important to determine the biological outcome of cell–cell interactions.

In this study, we investigated the influence of cytokine stimulation on the MHC class I and Ly49 membrane dynamics and density using fluorescence correlation spectroscopy (FCS) (20). It was previously shown that activating receptors diffuse in different patterns on educated versus uneducated NK cells, thus, coupling the diffusive pattern of receptors to NK cell functionality (21). In this paper, we found that despite the well-known upregulation of MHC class I by type I interferons (11, 12), the membrane density of mobile H-2D^d^ did not increase after cytokine stimulation. In line with this, there was no indication that the proportion of Ly49A receptors bound in *cis* increased after cytokine stimulation. Instead, we identified a subpopulation of NK cells that exhibited a particularly fast diffusion rate of both the inhibitory receptor Ly49A and of the MHC class I molecule H-2D^d^ upon cytokine stimulation. This is to our knowledge the first report on the effect of cytokines on molecular dynamics within the membrane of immune cells. The rapid diffusion may enable a faster decision process within the NK cell to kill or not to kill a target cell.

## Materials and Methods

### Experimental Animals

Mice expressing only the H-2D^d^ allele of MHC class I on C57BL/6 background were used. The generation of the mouse strain has been described previously (22). The mice were bred and maintained at the animal facility at the Department of Microbiology, Tumor and Cell biology, Karolinska Institutet, Sweden. The study was carried out in accordance to Swedish laws and regulations from Swedish board of agriculture (Jordbruksverket), approved by the northern Stockholm commission for ethics in experiments on animals, ethical permit N418-12.

### Cell isolation and Cytokine Stimulation

Natural killer cells were enriched from freshly isolated murine splenocytes by negative selection of all other cells, using MACS^TM^ NK cell isolation kit mouse II (Miltenyi Biotec Norden^AB^, Sweden) according to the manufacturer’s recommendations. After isolation, 2.5 × 10^6^ cells/ml were resuspended in alpha-MEM medium supplemented with 10 mM HEPES, 10% fetal bovine serum, 2 μM beta-mercaptoethanol, 100 μg/ml penicillin, and 100 μg/ml streptomycin. The cells were incubated at 37°C, 5% CO_2_ for 4 h, with or without cytokines. Cytokine concentrations were 1000 U/ml IL-2 (ImmunoTools, Germany) or 200 U/ml of each for IFN-α + β (Nordic BioSite, USA). In some experiments, cells were treated with 2 μg/ml Latrunculin B (Invitrogen, ThermoFisher Scientific, Sweden). Latrunculin B was added 15 min before measurement start, after cells were added to chambered glass slides to sediment (see below).

### Immunofluorescent Labeling and Flow Cytometry

Fc receptors were blocked using Innovex Fc blocker solution (Innovex Biosciences, Richmond, VA, USA). The cells were then surface stained with antibodies. For spectroscopy and microscopy, anti-H-2D^d^ (clone 34-5-8S) and anti-Ly49A (clone JR9.318) antibodies were used. The anti-H-2D^d^ antibody was conjugated with MFP488 (MoBiTec GmbH, Germany) or Abberior Star-635 (Abberior GmbH, Germany) for spectroscopy, or Alexa647^®^ dye (Life technologies, Sweden) for flow cytometry, according to the manufacturers’ protocols. The anti-Ly49A antibody was purified from hybridoma supernatants in our lab and conjugated to Abberior Star-635. Antibody conjugates were purified using PD minitrap G-25 kit column (GE healthcare Bioscience, Sweden). The 34-5-8S and JR9.318 antibodies do not affect the diffusion behavior of the investigated surface proteins (19). However, the 34-5-8S antibody breaks the *cis* interaction between H-2D^d^ and Ly49A (23). In flow cytometry measurements, anti-H-2D^d^ and anti-Ly49A antibodies were employed in different samples, so as to avoid artificially high levels of “free” Ly49A caused by the presence of the anti-H-2D^d^ antibody. Antibodies for flow cytometry were Ly49A (clone YE1/48.10.6)-PE, NK1.1-PerCP-Cy5.5, and CD3-FITC (Biolegend and BD Biosciences). Cells for flow cytometry were subsequently stained with Live/dead ^®^ fixable aqua dead cell stain kit 405 nm (Life Technologies, USA).

Flow cytometry data were acquired in a BD FACSCalibur^TM^ flow cytometer (BD Biosciences, San Jose, CA, USA) and analyzed in FlowJo (version 9.7). NK cells were gated on the CD3 negative and NK1.1 positive lymphocyte population, as determined by forward and side scatter. The percentage difference between controls incubated at 37°C without cytokines and cytokine-treated cells was calculated using mean fluorescence intensity (MFI).

### Fluorescence Correlation Spectroscopy

Fluorescence correlation spectroscopy measurements were performed using a Zeiss 510 microscope equipped with a Confocor 3 system (Carl Zeiss Microimaging GmbH), a 30 mW Argon-ion (488 nm), a 5-mW Helium–Neon (633 nm) laser, and a C-Apochromat 40x/1.2 NA water objective (24). The room temperature was 19°C. Calibration measurements of aqueous solutions of Alexa-647 (2 nM) and Rhodamine-110 (20 nM) with the laser excitation attenuated to 10, 5, 3, 1, and 0.5% of the maximal laser powers were first acquired. Cells were suspended in a 1:1 mixture of phosphate buffered saline and transparent RPMI medium, containing 0.5% fetal bovine serum. They were allowed to sediment in chambered cover glass slides (Fisher Scientific, Sweden) coated with Poly-l-Lysine (Sigma-Aldrich) for 20 min before experiment start. The sample identity (control, IL-2, and IFN-α + β stimulated) was blinded for the operator. The focus was positioned on the upper cell membrane. Only live (as judged by a distinct, rounded nucleus in the differential interference contrast image) Ly49A positive NK cells were selected for FCS measurements. Laser excitations for membrane measurements were 25–45 μW (representing 0.15–0.3% of maximal power for the 488 nm laser and 0.5–1% for the 633 nm laser). Five to seven 10-s repeats were measured per cell. Free antibodies in solution were measured with the same concentration and power as used for the cells, in non-stick-treated wells (PLL-PEG, Surface Solutions) to avoid depletion of antibodies from the solution.

### FCS Analysis

Autocorrelation curves were generated by the Confocor 3 software and further analyzed using a MATLAB-based algorithm with a graphical user interface for fitting (25, 26). Free dyes and antibodies were fitted with 3D diffusion with one diffusion component and one triplet state (27):

(1) $$\begin{array}{c} G_{3D}(\tau )=\frac{1}{N}*\;{\left(1+\frac{\tau }{\tau_{D}}\right)}^{-1}*\sqrt{\left(1+S^{-2}*\frac{\tau }{\tau_{D}}\right)}*\;\left(1+T1*{\left(1-T1\right)}^{-1}*exp\left(\frac{-\tau }{\tau_{T}}\right)\right)+1 \end{array}$$

Parameter definitions: *N* is the mean number of fluorescent entities within the focal volume, τ is the correlation time, τ_D_ is the average residence time in the focal detection volume (translational diffusion time), T1 is the average probability for the fluorophores to be in the triplet state, τ_T_ is the relaxation time for the singlet–triplet state transitions, and S denotes the ratio of height to waist diameter for the focal volume (axial to radial radii). The focal waist radius (ω) for the green and the red detection channels were calculated for each experimental day from the known diffusion coefficients of the free fluorophores, D; for Rhodamine-110, 3.3 × 10^−10^, and for Alexa-647, 4.4 × 10^−10^ m^2^/s (28, 29).

(2) $$\omega =\sqrt{\frac{\text{observed }\tau_{D}}{4*D}}$$

For cell measurements, individual repeats displaying bleaching, large clusters of bright entities, or cell movements, were removed. Remaining repeats were averaged and autocorrelation curves fitted. Due to the lack of *a priori* knowledge about any subpopulations of H-2D^d^ or Ly49A with different diffusion rates, the average diffusion rate per cell for each surface antigen was defined. Likewise, the underlying data did not contain enough information to fit models with different modes of movement (e.g., free diffusion versus constrained diffusion or active transport). Thus, all lateral molecular movement was considered to be diffusion. The molecular transit time within cell membranes was, thus, fitted by a 2D diffusion model with one diffusion component and one triplet state (28):

(3) $$G_{2D}(\tau )=\frac{1}{N}*{\left(1+\frac{\tau }{\tau_{D}}\right)}^{-1}*\left(1+T1*{\left(1-T1\right)}^{-1}*exp\;\left(\frac{-\tau }{\tau_{T}}\right)\right)+1$$

The diffusion coefficients of membrane-bound H-2D^d^ and Ly49A were obtained from the experimentally determined τ_D_ values (Eqs. 2 and 3). Densities of diffusing entities of H-2D^d^ and Ly49A were calculated from *N* from Eq. 3 and the focal waist radius ω for the individual color channels, using Eq. 4.

(4) $$\text{Density}=\frac{N}{\left(\omega^{2}*\pi \right)}$$

The molecular brightness, i.e., the detected fluorescence intensity per moving entity, was calculated from the overall detected photon count rate/*N*. To compensate for technical day-to-day variations, all cell and free antibody brightness values were adjusted based on the brightness of free fluorophores in the same experiment. To avoid a putative influence from background signal in cells where the antigens were expressed at a too low level, cells with a brightness of <33% of free antibodies were excluded from further analysis. For cells where one color channel was excluded, the other color channel was still used. Cells having a residence time τ_D_ >500 ms were also excluded from analysis. Such long τ_D_ could indicate movement of the whole cell, or membrane protrusions rather than movement of the individual molecules in the membrane.

### Antibody Binding Efficiency

To define the binding efficiencies of H-2D^d^ (clone 34-5-8S) and Ly49A (clone JR9.318) antibodies, transfected cell lines with GFP-coupled variants of the respective proteins were employed. For H-2D^d^, a previously described EL4-D^d^-GFP cell line was used (30). The Ly49a cDNA was amplified by polymerase chain reaction from primary mouse NK cells and cloned with a GTC GAC GGC AGC CAA AAA ACC linker downstream of GFP into the *Sal*I site of the MigR retroviral construct. For Ly49A binding efficiency, AA8-CHO cells were transfected with the EGFP–Ly49a construct according to the manufacturer’s protocol (Lipofectamine^®^2000, Invitrogen, Sweden). Both antibodies were labeled with Abberior star-635. Autocorrelation curves were measured and further analyzed as described above. Relative antibody binding efficiencies were determined by dividing the density of antibodies at the cell membrane with the density of labeled construct within the same measurement.

### Multivariate Analysis

The FCS dataset was investigated using the software SIMCA, version 14 (Umetrics AB, Umeå, Sweden). Only cells containing values for all H-2D^d^ and Ly49A variables (brightness, density, and diffusion) after application of threshold were used. Underlying trends in the data were investigated by principal component analysis (PCA) and orthogonal projections to latent structures and discriminant analysis (OPLS-DA) (31–33).

Principal component analysis is a projection method where data are analyzed without bias. Systematic variation in the data is summarized into latent variables, called scores (T). T1 is the direction in *N*-dimensional variable space summarizing the most of the systematic variation and T2 visualizes the second most variation. One individual PCA model was built per cell treatment group (data not shown). Outliers were detected using the Hotellings T2 ellipse, a two-dimensional representation of the 95% confidence interval. Cells outside of the Hotellings T2 ellipse were classified as outliers. Two outliers were identified for control, one for IL-2, and one for IFN-α + β and excluded from the subsequent OPLS-DA analysis.

Orthogonal projections to latent structures and discriminant analysis is similar to PCA in that it is a projection method, but it analyzes the data in relation to one selected Y variable (in our case, the classification of cells as control, IL-2 or IFN-α + β-stimulated) (33). All Y-related variation is visualized in the first “predictive” principal component, while subsequent “orthogonal” principal component(s) display variation unrelated to Y. In our model, only the first predictive component was significant.

### Cell Size Analysis

Overview confocal images were captured during the same sessions as FCS measurements. At least 11 z-sections of cells were captured, with the middle section of the z-stacks centered on the widest cellular diameter for the majority of cells. The image z-stacks were projected in ImageJ and analyzed in Cell Profiler using application-specific modules (34). Circular cell edges were identified and the cell area was calculated.

### Statistical Analysis

Statistical tests were performed in the software Prism (version 6.0, GraphPad, La Jolla, CA, USA). Outliers in the FCS data were identified using the Grubb’s test (35). If a cell was identified as an outlier in any variable for Ly49A or H-2D^d^, the entire cell was removed from further analysis for that color channel. D’Agostino–Pearson omnibus test for normal distribution was performed. The data were not normally distributed; however, transformation by the natural logarithm resulted in normally distributed populations for density and diffusion (36). The transformed data were, thus, further analyzed by one-way analysis of variance, ANOVA, and Holm–Sidak’s multiple comparison test. For brightness, H-2D^d^ in IL-2 stimulated cells, and Ly49A in IFN-α + β stimulated cells, natural logarithm transformation did not lead to normality. Brightness of cytokine-treated cells was, therefore, compared to the control by Kruskal–Wallis one-way ANOVA and Dunn’s multiple comparisons test. The same test was also applied for the cell size. The geometric mean and 95% confidence intervals were calculated (36). For flow cytometry data, upregulation of Ly49A and H-2D^d^ expression after cytokine stimulation was assessed by one-way ANOVA, Dunn’s multiple comparisons test on selected pairs of variables. *P*-values of 0.05 < *p* < 0.01, 0.01 < *p* < 0.001, and *p* < 0.001 were considered statistically significant (noted as *, **, and *** in figures).

## Results

### Cytokine-Stimulated NK Cells Exhibit Faster Diffusion Dynamics of H-2D^d^ and Ly49A at the Plasma Membrane

**Figure 1:**
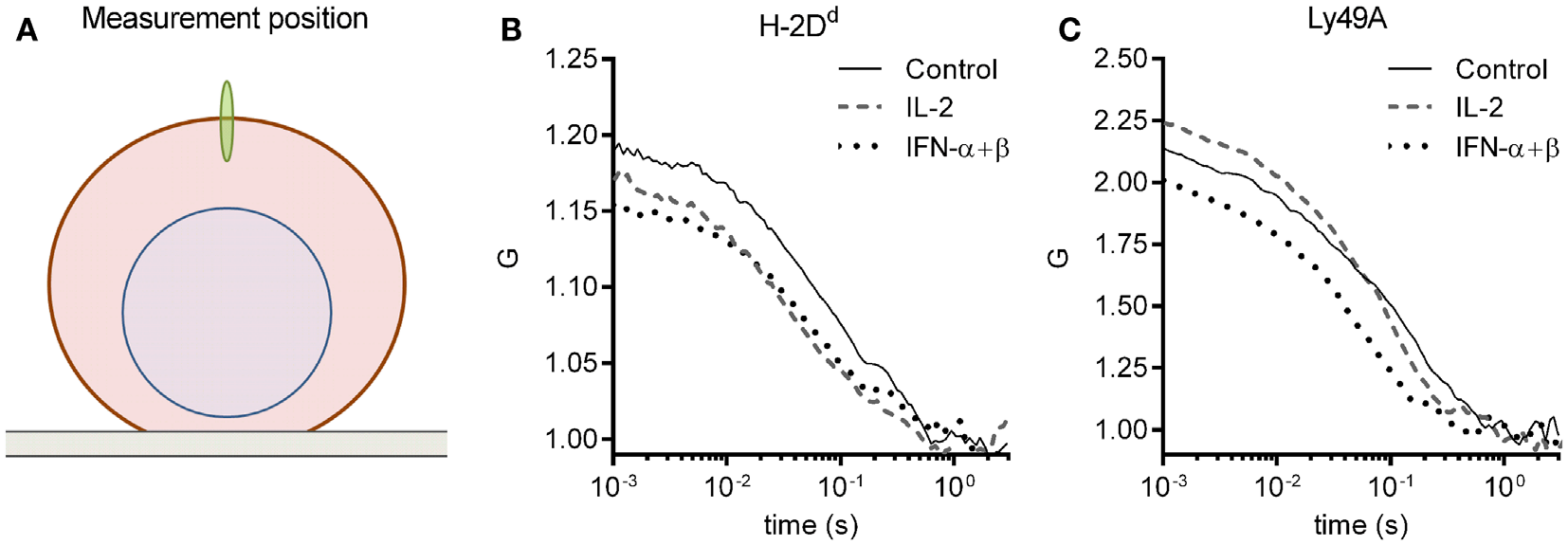
**FCS measurement and representative autocorrelation curves**. The autocorrelation curves of H-2D^d^ and Ly49A were measured in freshly isolated murine NK cells after 4 h of cytokine stimulation. **(A)** A schematic illustration of the positioning of the focus (not to scale). FCS measurements were performed on the upper cell membrane. **(B,C)** Representative FCS autocorrelation curves for a control, an IL-2, and an IFN-α + β stimulated cell for **(B)** H-2D^d^ entities, and **(C)** Ly49A entities. The cells were selected based on a location close to the center in the multivariate PCA analysis for each group (see Materials and Methods for description of the PCA analysis).

The molecular density and diffusion coefficients are important parameters for the rate of molecular interactions, and thus for subsequent biological outcomes. FCS measures molecular diffusion rates with high accuracy, and give an estimate of molecular densities, also within the plasma membrane of cells (28). We, therefore, investigated the impact of short-term cytokine stimulation on the molecular dynamics within the plasma membrane of the inhibitory receptor Ly49A, and its ligand, H-2D^d^, using FCS. Freshly isolated NK cells from mice expressing only the H-2D^d^ allele of MHC class I were used. All NK cells were thus educated. NK cells were enriched by negative selection to a percentage of 85 ± 5% (data not shown). The cells were thereafter cultured for 4 h, either with IL-2 or IFN-α + β, or without cytokines. H-2D^d^ and Ly49A were each labeled with antibodies that have been used previously for FCS measurements, and do not induce dimerization or affect the diffusion behavior in any other detectable fashion (19). FCS measurements were performed on the top of the cell membrane (Figure 1A). A series of autocorrelation curves were generated and averaged for each NK cell, and then fitted to Eq. 3. Representative curves from each stimulation group are shown in Figures 1B,C.

**Figure 2:**
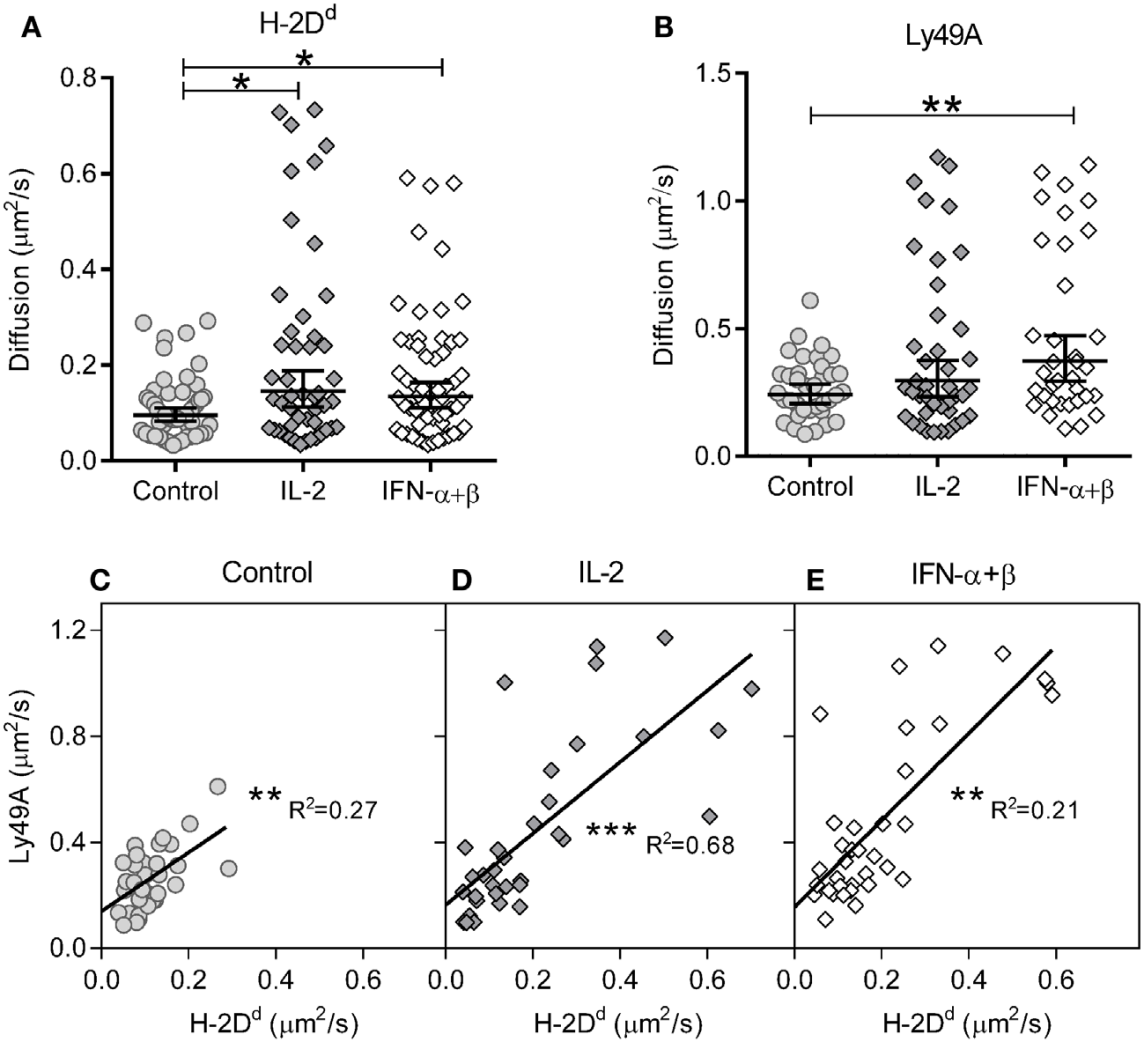
**Diffusion rates of H-2D^d^ and Ly49A are increased after 4 h of cytokine stimulation**. The diffusion rates were measured in freshly isolated murine NK cells after 4 h of cytokine stimulation. **(A)** Diffusion rates of H-2D^d^ in individual cells. **(B)** Diffusion rates of Ly49A. Cells in A and B are the same as in Table 1. The lines show Geometric mean, error bars show 95% confidence interval. **(C–E)** Linear regression of Ly49A and H-2D^d^ diffusion coefficients in the same cells, 35, 32, and 33 cells for control, IL-2, and IFN-α + β, respectively. **(C)** Diffusion rate in control cells. **(D)** Diffusion rate in IL-2 stimulated cells. **(E)** Diffusion rate in IFN-α + β stimulated cells. * = 0.05 < *p* < 0.01, ** = 0.01 < *p* < 0.001, *** = *p* < 0.001 in A, B compared to control, in C–E for slope difference compared to 0. R^2^, least squares goodness of fit.

We first investigated the impact of cytokine stimulation on the diffusion rate. H-2D^d^ diffused rather slowly in resting cells (Figure 2A; Table 1). Interestingly, H-2D^d^ diffused significantly faster on both IL-2 and IFN-α + β stimulated NK cells, compared to control cells (Figure 2A; Table 1). The diffusion of Ly49A was significantly faster than that of H-2D^d^ in resting cells and, similarly to H-2D^d^, increased significantly on IFN-α + β stimulated NK cells (Figure 2B; Table 1). Notably, in addition to a slight shift of the whole population toward faster diffusion, several cells in both stimulated NK cell populations exhibited a markedly faster diffusion, compared to any of the control cells (Figures 2A,B). The diffusion rate of H-2D^d^ co-varied significantly with that of Ly49A in the same cell, in both control and cytokine-stimulated cells (Figures 2C–E). Thus, some, but not all, NK cells exhibited a markedly faster diffusion of both H-2D^d^ and Ly49A after stimulation with either IL-2 or IFN-α + β.

**Table 1:** **Density, brightness, and diffusion rate obtained from FCS analysis**.

Values from FCS		Control L95, **GM**, U95	IL-2 L95, **GM**, U95	IFN-α **+** **β** L95, **GM**, U95
Density (N/μm^2^)	H-2D^d^	43.3, **50.5**, 58.9	35.6, **42.4**, 50.4	45.6, **54.5**, 65.0
	Ly49A	3.46, **4.21**, 5.11	3.04, **3.79**, 4.72	2.78, **3.47**, 4.35
Brightness (kHz)	H-2D^d^	0.84, **0.99**, 1.18	1.17, **1.34**, 1.54	0.92, **1.10**, 1.32
	Ly49A	7.0, **8.7**, 10.7	8.4, **10.5**, 13.0	7.9, **10.2**, 13.2
Diffusion coefficient (μm^2^/s)	H-2D^d^	0.082, **0.095**, 0.110	0.112, **0.145**, 0.188	0.110, **0.135**, 0.164
	Ly49A	0.207, **0.242**, 0.283	0.233, **0.296**, 0.376	0.294, **0.373**, 0.472

*The bold is to highlight the geometrical mean, in contrast to the confidence interval limits. L95, lower 95% confidence interval limit. GM, geometric mean. U95, upper 95% confidence interval limit. Geometric mean and 95% confidence interval limits calculated from data transformed by the natural logarithm, and thereafter back-calculated to raw data format. Values are not corrected for antibody brightness and binding efficiency. H-2D^d^ = 55, 48, and 58 cells, Ly49A = 36, 41, and 35 cells, for control, IL-2, and IFN-α + β, respectively. Data pooled from seven independent experiments*.

The reason for the relatively slow molecular motion of H-2D^d^ in resting NK cells could be either that the molecules are specifically interacting with other proteins, or that the diffusion is physically restricted. This restriction could in turn be due either to crowding, if the membrane is densely populated with proteins, or confinement by the cytoskeleton. To investigate the role of the cytoskeleton, we treated NK cells with Latrunculin B, which disrupts the actin cytoskeleton. Latrunculin B had no effect on the diffusion rate of neither H-2D^d^ nor Ly49A on resting NK cells. After stimulation with either IFN-α + β or IL-2, there was a tendency of faster movement of Ly49A, albeit not statistically significant (Figure S1 in Supplementary Material). This suggested that the slow movement of H-2D^d^ is due either to crowding or to interaction with other molecules.

### Negligible Changes in Density of Diffusing H-2D^d^ and Ly49A upon Cytokine Stimulation Despite Increased Total Cell Surface Expression

**Figure 3:**
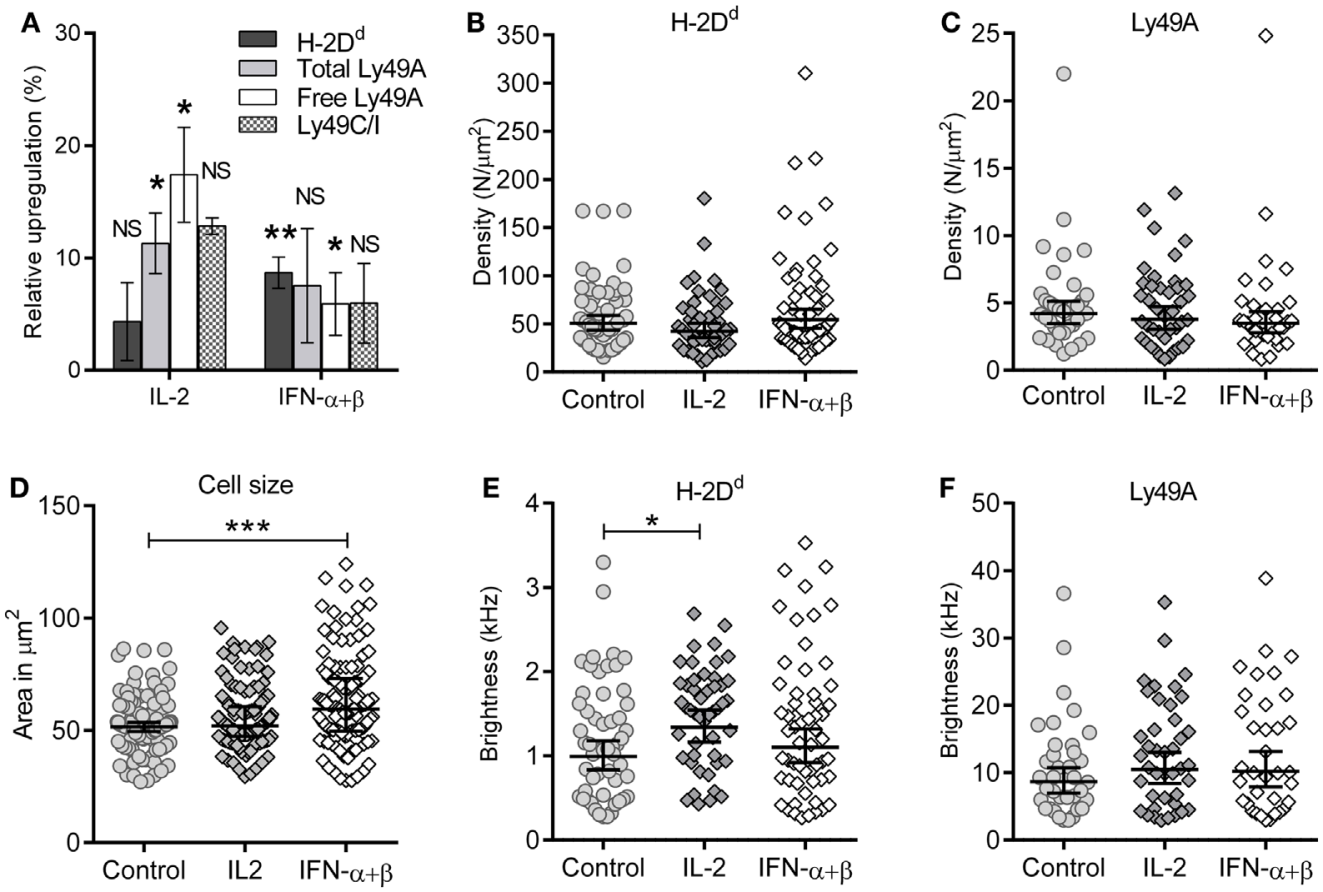
**Maintained membrane density despite upregulation of H-2D^d^ and Ly49A at the cellular level**. **(A)** Expression of cell markers: H-2D^d^, “total” Ly49A, Ly49C/I, and “free” Ly49A (not *cis*-bound), as measured by flow cytometry after 4 h of cytokine stimulation. Percentage upregulation calculated with respect to a 37°C incubated control without any cytokines. The difference in upregulation of “free” Ly49A between IL-2 and IFN-α + β stimulation was statistically significant (*p* = 0.02), other differences between the two cytokines were non-significant. Significances were tested by one-way ANOVA, Dunn’s multiple comparisons test on selected pairs of variables. Bar graph represents mean ± standard error of the mean (*n* = 8 for H-2D^d^, *n* = 7 for Ly49A total and free, *n* = 2 for Ly49AC/I). **(B,C)** Density of diffusing entities measured by FCS; **(B)** H-2D^d^ entities and **(C)** Ly49A entities. The lines show Geometric mean, error bars = 95% confidence interval. **(D)** Cell cross-sectional area of individual cells at their widest point. The geometric mean of the cell area at the widest point determined for control was 52.8 μm^2^ (95% confidence interval limit (CI) 40.7–54.9 μm^2^), for IL-2 55.2 μm^2^ (CI 52.9–57.5 μm^2^, and for IFN-α + β 62.5 μm^2^ (CI 59.0–66.0 μm^2^). 127, 132, and 130 cells, for control, IL-2, and IFN-α + β, respectively. **(E)** Brightness per H-2D^d^ entity. **(F)** Brightness per Ly49A entity. The cells in B, C, E, and F are the same as in Table 1. **(C–F)** Bar and error = Geometric mean with 95% confidence interval. NS, not significant. * = 0.05 < *p* < 0.01, ** = 0.01 < *p* < 0.001, *** = *p* < 0.001.

Type I interferons are known to upregulate MHC class I on most cell types. We investigated the overall surface expression of Ly49A and H-2D^d^ per NK cell after cytokine stimulation, using flow cytometry. NK cells were isolated and stimulated as for the FCS experiments. H-2D^d^ was modestly but significantly upregulated on Ly49A^+^ NK cells by IFN-α + β stimulation, but not by IL-2 (Figure 3A, dark gray bars). Ly49A was upregulated by IL-2, while there was no statistically significant difference after IFN-α + β stimulation (Figure 3A, light gray bars). Ly49C/I, which has the MHC class I allele H-2K^b^ as ligand, was upregulated to approximately the same extent (Figure 3A, checkered bars). Thus, both a Ly49 receptor with and one without a specific MHC class I ligand in the host were upregulated at the cellular level. The mRNA levels of H-2D^d^ in NK cells increased after both types of cytokine stimulation already after 2 h: 1.4 times increase for IL-2 and 1.8 times increase for IFN-α + β (data not shown).

The molecular density is, however, a more relevant parameter for the rate of molecular interactions than the total expression level per cell. Stimulation with these cytokines did not result in increased densities of neither H-2D^d^ nor Ly49A at the cell surface (Figures 3B,C; Table 1).

A potential reason for overall increase in protein expression while maintaining density would be a simultaneous increase of the overall cell membrane area. We, thus, determined the average cross-sectional area of stimulated and unstimulated NK cells. By acquiring Z-stacked 2D overview confocal images of several cells per image, the widest point of each cell was identified. The cross-sectional area increased significantly after 4 h of IFN-α + β stimulation, but not by IL-2 stimulation (Figure 3D). Thus, the increased cell size, rather than an increased cell surface density, could account for the increased expression level per cell of H-2D^d^ after IFN-α + β stimulation.

Fluorescence correlation spectroscopy is not capable of detecting single molecules if they are diffusing as a complex. An analysis of the brightness of moving entities can instead give an indication of whether more than one molecule diffuse together. H-2D^d^ displayed a slightly but significantly higher brightness after IL-2 stimulation (Figure 3E; Table 1). This could potentially indicate a higher degree of homo-clustering. No significant differences in the brightness were observed for Ly49A (Figure 3F). The average brightness of freely diffusing antibodies in solution was 0.82 ± 0.12 kHz for the H-2D^d^ and 8.2 ± 0.53 kHz for the Ly49A antibody. These values were somewhat lower than for the entities diffusing in the membrane (Table 1). However, only H-2D^d^ in IL-2 stimulated cells were significantly brighter than the free antibody (*p* < 0.05), strengthening the conclusion of a clustering in this situation. An increased clustering of H-2D^d^ goes in line with the slightly higher fluorescence intensity per cell detected by flow cytometry (Figure 3A, not statistically significant).

In conclusion, the early H-2D^d^ and Ly49A upregulation in NK cells by IFN-α + β correlate with an increased cell size, rather than a higher cell surface density. An increased clustering of H-2D^d^ was indicated after IL-2 stimulation. The increased Ly49A expression after IL-2 stimulation at the cellular level was, however, not explained, neither by an increased density of diffusing Ly49A, nor an increased cross-sectional area of the cell.

To estimate the actual density of diffusing H-2D^d^ and Ly49A entities, we next determined the binding efficiency of the H-2D^d^ and Ly49A antibodies using transfected cell lines expressing GFP-coupled versions of the respective antigens. Fluorescent antibodies labeled 18.3 ± 5.6% of GFP-coupled H-2D^d^ and 10 ± 5.9% of GFP-coupled Ly49A expressed on the cell membrane (data not shown). Thus, both with and without correcting for binding efficiency, H-2D^d^ was more densely expressed compared to the Ly49A receptor on the membrane of resting NK cells, which goes well in line with previous reports that most Ly49A receptors are bound in *cis* on H-2D^d^ expressing NK cells (37, 38).

### The Total Amount of Ly49A Receptors Able to Interact in *trans* Increase by IL-2 Stimulation

For detection of Ly49A in the FCS experiments, we used an antibody clone (JR9.318), which detects both free and *cis*-bound Ly49A (37, 39). The total levels of Ly49A per cell could, therefore, be measured. However, if the fraction of *cis*-bound receptors changed, that would not be detected. The relative amount of free Ly49A at the cellular level was, thus, estimated by another antibody clone (YE1/48). This antibody binds less efficiently to Ly49A that is bound to H-2D^d^ in *cis* (37). Also when using this antibody, the amount of detected Ly49A was significantly increased after IL-2 stimulation. There was also a smaller, albeit statistically significant upregulation in the IFN-α + β stimulated cells (Figure 3A). Thus, IFN-α + β stimulation did not lead to a decrease of free Ly49A receptors, despite the previously well-established upregulation of MHC class I by interferons, and after IL-2 stimulation, there was a substantial increase in the amount of Ly49A receptors free to interact with H-2D^d^ in *trans*. For the IFN-α + β stimulation, this was in line with the negligible changes in cell surface density of both molecules, as observed by FCS.

### Cytokine-Stimulated NK Cells Contain a Subpopulation of Cells with Rapid Diffusion Dynamics of H-2D^d^ and Ly49A on the Plasma Membrane

To further characterize differences between control, IL-2, and IFN-α + β stimulated cells, a multivariate analysis was performed on the FCS dataset. All six available variables were employed: the diffusion rate, density, and brightness of Ly49A and H-2D^d^, respectively. Only cells with no missing data were used (see Material and Methods for exclusion criteria).

**Figure 4:**
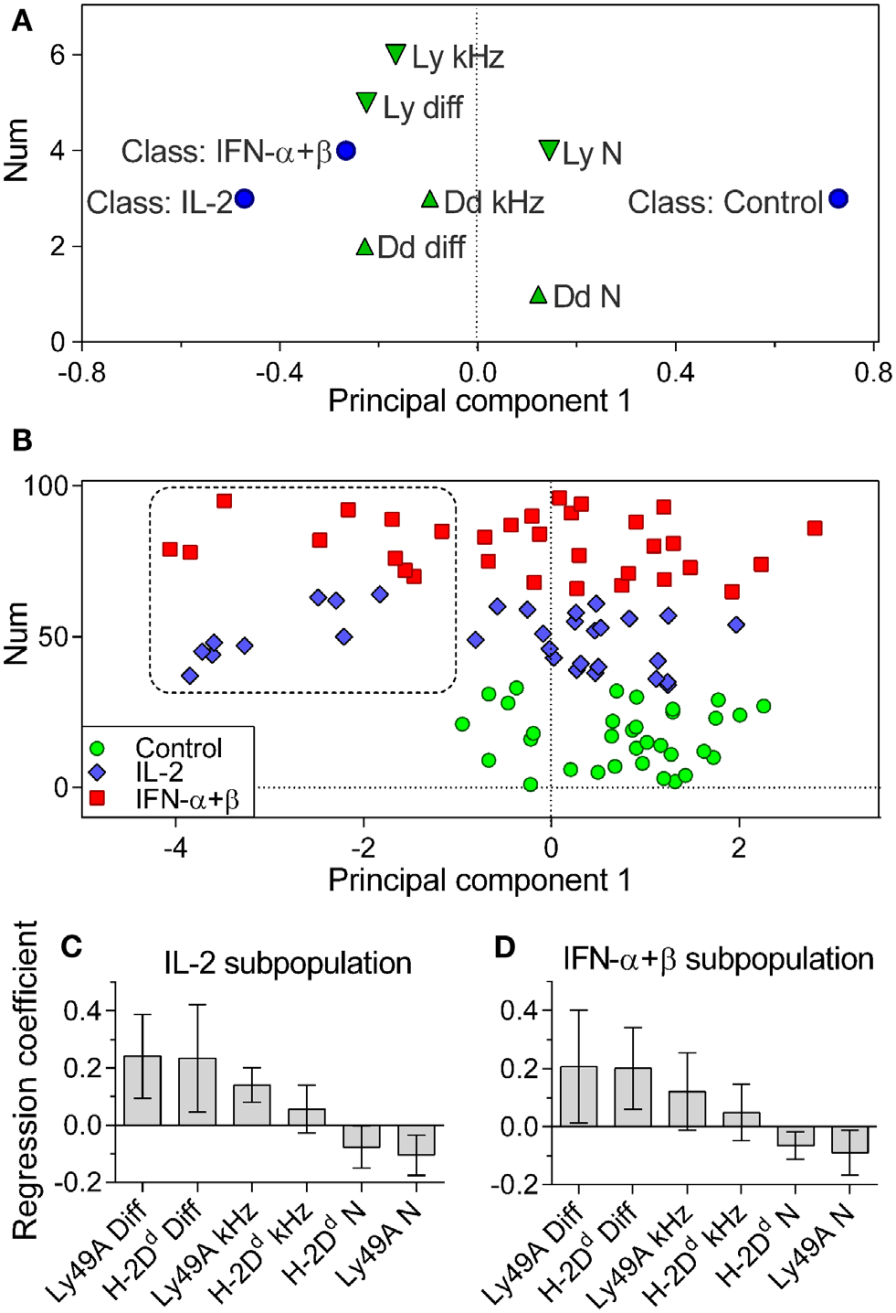
**Cytokine-stimulated cells contain a subpopulation of cells with rapidly diffusing entities**. Multivariate OPLS-DA analysis of the FCS data. **(A)** Classes: Control, IL-2, and IFN-α + β stimulated cells (circles). Variables contributing to the separation: downward pointing triangles = Ly49A variables, upward pointing triangles = H-2D^d^ variables. Variables close in space to the class are positively correlated to that class. **(B)** The positioning of individual cells and the identification of a subpopulation of cytokine-treated cells, not overlapping with control cells. Circles, control cells; diamonds, IL-2 stimulated cells; squares, IFN-α + β-stimulated cells. Vertical axis label “Num” refers to the order of the values in the dataset and has no significance for the model. **(C)** Variables characterizing cells in the IL-2 subpopulation. **(D)** Variables characterizing cells in the IFN-α + β subpopulation. Diff, diffusion rate; kHz, brightness; N, density. Error bars in C and D represent the 95% confidence interval. The model was built only on cells where all variables were present for both Ly49A and H-2D^d^: 33, 31, and 32 cells for Control, IL-2, and IFN-α + β, respectively. Cells pooled from seven independent experiments.

Control, IL-2, and IFN-α + β stimulated cells were assigned to individual classes. Differences between these classes were investigated by discriminant analysis (OPLS-DA). The OPLS-DA model could explain 41.3% of the variation between classes, 6.5% of the variation unrelated to class separation, and predicted 3.6% of the variation in the data. Control cells were significantly separated from cytokine-stimulated cells (Figure 4A). IL-2 and IFN-α + β-stimulated cells could not be separated from each other with statistical significance. This is visualized by their class-characterizing variables being close in variable space (Figure 4A). In the multivariate analysis, it became apparent that a subpopulation among the cytokine-stimulated cells was distinctly separated from the control cells (Figure 4B). In the global data set, 29% of the IL-2 and 31% of the IFN-α + β stimulated cells belonged to this subpopulation. However, for the IFN-α + β-stimulated cells, five out of the seven experiments contributed to the subpopulation, whereas the IL-2 subpopulation only came from three out of the seven experiments. IFN-α + β stimulation, thus, led to a more robust appearance of the discrete subpopulation.

We investigated which variables characterized the subpopulation among the IL-2 and IFN-α + β-stimulated cells. The two main defining variables were fast diffusion of H-2D^d^ and Ly49A for both IL-2 and IFN-α + β-stimulated cells (Figures 4C,D). The IL-2-stimulated subpopulation was further characterized by a high brightness and low density of Ly49A (Figure 4C). A higher degree of clustering of Ly49A was, thus, indicated in the fast subpopulation after IL-2 stimulation. The IFN-α + β fast subpopulation displayed a low density of both H-2D^d^ and Ly49A, without statistically significant changes in the brightness (Figure 4D). This could be reflecting a dilution of molecules due to an increased cell size (Figure 3D).

Conclusively, both types of cytokine stimulations resulted in the emergence of a large subpopulation of NK cells separated from the other cells by a significantly faster diffusion rate of both H-2D^d^ and Ly49A within the cell membrane. After IL-2 stimulation, there was also an indication of clustering of Ly49 receptors in the subpopulation.

## Discussion

The most marked early impact of cytokine stimulation in this study was an increase in the diffusion rates of both investigated molecules. To our knowledge, this is the first time that an effect of cytokine stimulation on the molecular motion within the membrane of any naïve immune cell has been observed. Cytokines are major controllers of immune reactions, and diffusion rates are vital regulators of the molecular interactions leading to such reactions. Yet, little is known about how molecular dynamics are affected by cytokine stimulation. The concept of spatiotemporal dimensions at the molecular level in immune cell regulation is relatively new and has become available to study by the development of new fluorescence-based techniques. The principle of induction of faster diffusion of receptors and other immunologically relevant proteins by cytokines may expand to other receptors, immune cells, and cytokines. The full scope of how cytokines affect molecular dynamics, and the biological relevance, is not known at this point. Given the large importance of cytokine stimulation in the immune system, this aspect of cytokine regulation should be further investigated. One example of a situation where cytokine stimulation is important is in NK cell activation to produce more potent killers as immunotherapy for malignant diseases (40).

Ly49A interactions with MHC class I on target cells are essential for the early part of synapse formation and receptor signal integration in the interface between NK cells and target cells (41). The amount of Ly49 receptors bound in *cis* was previously shown to be a diffusion-limited reaction, in similarity to most other protein–protein interactions (19). Ly49 binding of MHC class I in *cis* and *trans* uses the same binding site and the affinity of the two interactions, thus, ought to be in the same range (17). It is, thus, likely that the diffusion rate also affects how fast the Ly49A receptor find a steady state in the binding to MHC class I in *trans*. The higher mobility of H-2D^d^ and Ly49A could, thus, potentially enable a more rapid formation of an (inhibitory) immunological synapse, and thus a faster decision process whether a target cell is abnormal or not.

It has been observed that a minority of the IL-2-stimulated NK cells perform the majority of kills after several days of IL-2 stimulation (8). Our observation of increased molecular dynamics within a subpopulation could, thus, tentatively be an early sign of this functional heterogeneity within the NK cell population. The subpopulation did not correspond directly to NK cells upregulating CD69 or ICAM-1, since they did not exhibit a similar upregulation pattern on the NK cell population level (data not shown). A difference in molecular dynamics between educated and uneducated NK cells has previously been shown (21). However, since in our case only Ly49A^+^ NK cells from H-2D^d+^ mice were investigated, all NK cells were educated. Therefore, the heterogenic response could not be explained by a difference in educational status. The fact that the size of the subpopulation differed between experiments suggests that environmental factors in the individual animal and/or the experimental conditions affects how many of the NK cells will respond to cytokine stimulation with a faster molecular movement. It also suggests that a relatively large fraction of the NK cells have the potential to respond with faster molecular dynamics, if stimulated appropriately.

The fact that both H-2D^d^ and Ly49A moved faster in the same cells after cytokine stimulation could potentially indicate a general change in the cell membrane dynamics. This could at least partly be due to an increased cell surface area, making the proteins in the cell membrane overall less crowded. A crowding effect is not unlikely, given the high density at which H-2D^d^ was expressed in this study. H-2D^d^ alone was expressed at around 50 molecules/μm^2^, and crowding occurs starting from around 200 proteins/μm^2^ expressed in total (19, 42). Crowding in resting NK cells was further supported by the lack of effect of actin cytoskeleton disruption, despite the fact that diffusion of MHC class I has previously been reported to be restricted by the cytoskeleton (43–47). By contrast, there was a tendency of confinement by the cytoskeleton in cytokine-stimulated cells, which would go well in line with a release from crowding by dilution of other surface proteins as a consequence of increased cell size, making the restriction by the cytoskeleton more apparent. Resting murine NK cells, thus, seem to have a membrane densely populated with protein, but ready to within a few hours expand in size and simultaneously also alter their membrane dynamics.

Potential other underlying mechanisms for an increased diffusion rate include alterations of *cis* interactions with other binding partners, such as other Ly49 receptors or ICAM-1, or alterations in the actin cytoskeleton structure, both of which have been shown to be affected by cytokine stimulation (47–50). A faster general diffusion could also tentatively be a reflection of a higher cellular metabolism (51) or a change in the mode of molecular movement, from passive diffusion to active transport.

The diffusion of MHC class I has been studied in a variety of murine and human cell lines. Recorded diffusion coefficients by FCS and fluorescence recovery after photobleaching (FRAP) ranged from 0.01 to 0.9 μm^2^/s (19, 43, 45, 46, 52, 53). Single particle tracking gave rise to diffusion coefficients down to 10 times slower than those detected from FRAP and FCS (45). While all previous studies on MHC class I diffusion were carried out in cell lines, we studied the diffusion in resting primary cells. Considering that most cell lines are replicating and, thus, more active than a resting cell, the fact that the H-2D^d^ diffusion coefficient of 0.095 μm^2^/s fits within the range of previously published data is worth to note.

The diffusion of Ly49A was always at least twice as fast as that of H-2D^d^. This was surprising; especially since many of the Ly49A receptors interact in *cis* with H-2D^d^. The Ly49A diffusion rate was similar to the human inhibitory receptor KIR2DL1 in a cell line system (0.23 ± 0.06 μm^2^/s) (54). It is possible that the relatively small fraction of H-2D^d^ and Ly49A that were bound in *cis* in this study did not make an impact on the average diffusion rate observed. In the FCS part of this study, no detected H-2D^d^ was bound in *cis* to Ly49A, since the antibody used to detect H-2D^d^ in the FCS experiments prevents *cis* interaction (23). The detected Ly49A molecules would be a mixture of free and *cis*-bound to H-2D^d^, since the H-2D^d^ antibody binding efficiency was only around 19%. The difference in diffusion rate could, thus, be a result of hindered diffusion of H-2D^d^ molecules, in line with what has been reported in previous studies (43–47). The diffusion of H-2D^d^ could putatively also be hindered by clustering to itself or other partners, as discussed above. Different localization with regard to nanodomains, or a greater potential for active transport for Ly49A are other possible explanations for the observed differences.

The amount of “free” inhibitory receptors available to interact in *trans* increased substantially upon IL-2 stimulation. A much smaller increase of free inhibitory receptors was observed after IFN-α + β stimulation. The difference between the two cytokines in inducing upregulation of free Ly49A was statistically significant (Figure 3A). The more substantial upregulation of MHC class I induced by IFN-α + β at the cellular level could be one reason for the relatively smaller upregulation of free Ly49A receptors since more of the receptors could be bound in *cis* to H-2D^d^. The increased clustering of H-2D^d^ molecules after IL-2 stimulation (Figure 3E) could be another explanation contributing to the lower fraction of Ly49A receptors bound in *cis* observed by flow cytometry, if homo-clustering of MHC class I excludes binding of Ly49A in *cis*. For the IL-2 stimulation, the FCS and flow cytometry results are not completely concordant, as flow cytometry indicated an increase of total Ly49A expression that is still unexplained. A less rounded cell shape, giving a higher total cell surface area without increasing the cross-sectional area, or increased membrane ruffling, could potentially be explanations for this discrepancy. Another reason could be a higher fraction of immobile Ly49A, which would not be detected by FCS.

Even though FCS is a quantitative technique, exact concentrations obtained from FCS data should be regarded with some caution. Since FCS is based on intensity fluctuations, molecules have to be reasonably mobile to be detected. Immobile fractions, as well as bleaching, can contribute to under-estimation of the number of molecules (55). Putative error sources have been listed previously, and the total maximal error in the density determination was estimated to be around 40% (19). Given that the measurement conditions were very similar, this estimation is likely relevant also for this study. Since the error sources are in most cases stable throughout the experiments, the error in relative density between samples should, however, be minor, provided that the immobile fractions do not change. The determination of diffusion rates is also more robust than the density determination, even though also the diffusion rate can be over-estimated in case of bleaching.

Taken together, the heterogenic response to cytokine stimulation within the NK cell population shows that more than one type of response is induced in cytokine-treated cells, indicating subgroups within the NK cell population with different molecular dynamics. These differences are apparent already after 4 h of stimulation. The faster diffusion of inhibitory receptors could influence the rate of accessibility of receptors to target cell interactions, finally making cytokine-stimulated NK cells more efficient in screening of putative target cells. Studies further characterizing these subpopulations, identifying how they functionally differ from other stimulated NK cells, will tell what role this heterogeneity plays in the immune response against tumorigenic cells and infection.

## Author Contributions

SB-S and ES designed, planned and performed the FCS experiments in Figures 1–3, prepared figures, and wrote the manuscript. SB-S planned, performed and analyzed the FACS experiments in Figure 3A and analyzed the cell size data in Figure 3D. ES analyzed the FCS data and performed the multivariate analysis in Figure 4. AJS and TK performed the antibody labeling efficiency experiment, and commented on the manuscript. TS wrote the software to analyze the FCS experiments. JW provided feedback on the experimental design, analysis, and the manuscript. SJ designed experiments, analyzed data, provided mice and reagents, and wrote the manuscript. All authors reviewed the results and approved the final version of the manuscript.

## Conflict of Interest Statement

The authors declare that the research was conducted in the absence of any commercial or financial relationships that could be construed as a potential conflict of interest.

## Abbreviations

FCS, fluorescence correlation spectroscopy; OPLS-DA, orthogonal projections to latent structures, with discriminant analysis; PCA, principal component analysis.
