# Supplementary material for: Cytokines Induce Faster Membrane Diffusion of MHC Class I and the Ly49A Receptor in a Subpopulation of Natural Killer Cells
Source: Front Immunol. 2016 Feb 4;7:16. doi: 10.3389/fimmu.2016.00016 (PMC4740373; doi:10.3389/fimmu.2016.00016)
Supplement: Supplementary file 1 [file datasheet_1.docx]

Supplementary Material

Cytokines induce faster membrane diffusion of MHC class I and the Ly49A receptor in a subpopulation of Natural Killer cells

**Sunitha Bagawath-Singh, Elina Staaf, Arie J****an Stoppelenburg, Thiemo Spielmann, Taku Kambayashi, Jerker Widengren, Sofia Johansson^*^**

*** Correspondence:** Corresponding Author: sofia.e.johansson@ki.se

## Supplementary Figures


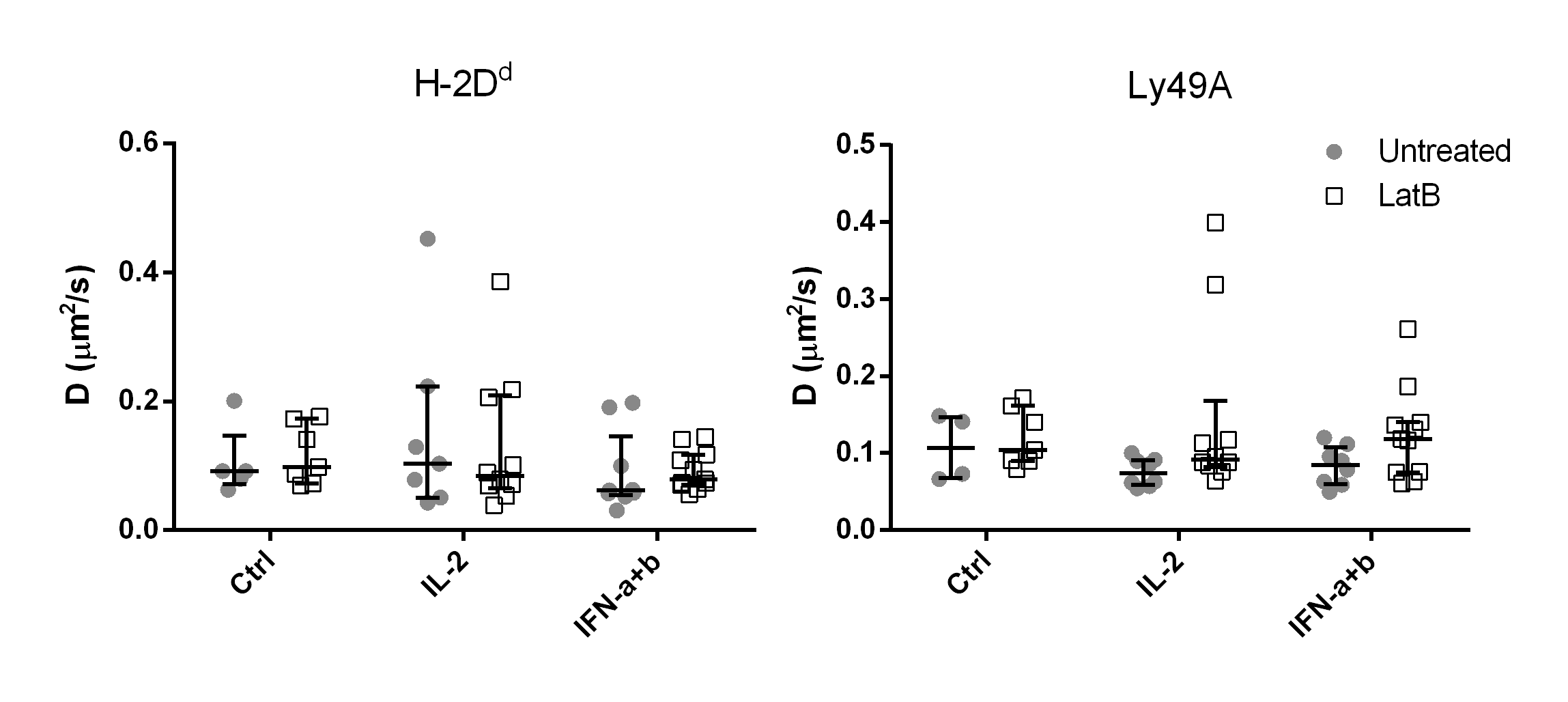


**Supplementary Figure 1.** Actin cytoskeleton disruption does not alter the diffusion rate of H-2D^d^ or Ly49A significantly. Freshly isolated NK cells were stimulated with IL-2 or IFN-a+b for four hours, or left unstimulated. Fifteen minutes before measurement start of each sample, cells were treated with Latrunculin B, or left untreated. The figure shows a compilation of two independent experiments with 5-11 cells per group. The bars show median with interquartile range.
